# Population-Based Screening for Functional Disability in Older Adults

**DOI:** 10.1093/geroni/igaa065

**Published:** 2020-12-22

**Authors:** Claire K Ankuda, Vicki A Freedman, Kenneth E Covinsky, Amy S Kelley

**Affiliations:** 1 Department of Geriatrics and Palliative Medicine, Icahn School of Medicine at Mount Sinai, New York, New York, USA; 2 Institute for Social Research, University of Michigan, Ann Arbor, USA; 3 Division of Geriatrics, University of California, San Francisco, USA; 4 Division of Geriatrics, Veterans Affairs Medical Center, San Francisco, California, USA; 5 Geriatric Research Education and Clinical Center, James J. Peters Veterans Affairs Medical Center, Bronx, New York, USA

**Keywords:** Aging, Functional assessment, Health services research, Public health

## Abstract

**Background and Objectives:**

Screening for functional disability is a promising strategy to identify high-need older adults. We compare 2 disability measures, activities of daily living (ADLs), and life space constriction (LSC), in predicting hospitalization and mortality in older adults.

**Research Design and Methods:**

We used the nationally representative National Health and Aging Trends Study of 30,885 observations of adults aged 65 years and older. Outcomes were 1-year mortality and hospitalization. Predictors were ADLs (receiving help with bathing, eating, dressing, toileting, getting out of bed, walking inside) and LSC (frequency of leaving home).

**Results:**

Of respondents, 12.4% reported 3 or more ADLs and 10.8% reported rarely/never leaving home. ADL disability and LSC predicted high rates of 1-year mortality and hospitalization: of those with 3 or more ADLs, 46.4% died and 41.0% were hospitalized; of those who never/rarely left home, 40.7% died and 37.0% were hospitalized. Of those with *both* 3 or more ADLs and who never/rarely left home, 58.4% died. ADL and LSC disability combined was more predictive of 1-year mortality and hospitalization than either measure alone. ADL disability and LSC screens identified overlapping but distinct populations. LSC identified more women (72.6% vs 63.8% with ADL disability), more people who live alone (40.7% vs 30.7%), fewer who were White (71.7% vs 76.2%) with cancer (27.6% vs 32.4), and reported pain (67.1% vs 70.0%).

**Discussion and Implications:**

LSC and ADLs both independently predicted mortality and hospitalization but using both screens was most predictive. Routine screening for ADLs and LSC could help health systems identify those at high risk for mortality and health care use.

**Translational Significance:** Using two distinct screens for functional disability, activities of daily living and life space construction, can help health systems and communities identify older adults at highest risk of death and hospitalization and appropriately target resources to better support them in the community.

Older adults with functional decline are at a high risk of poor health care outcomes. After new onset of disability in activities of daily living (ADLs; receiving help with bathing, toileting, walking, eating, getting in/out of bed and dressing), adults over the age of 65 have a 56% 2-year mortality rate (Ankuda et al., 2020). Indeed, disability predicts mortality more accurately than multimorbidity (Landi et al., 2010) and severe medical conditions alone (Kelley & Bollens-Lund, 2018; Kelley et al., 2017).

The impact of functional decline on burdensome, high-cost, health care use has come to the attention of health system leaders. Functional disability increases the likelihood of hospital readmissions (Greysen et al., 2015), overall health care costs (Aldridge & Kelley, 2014; Chan et al., 2002), and health care costs specifically at the end of life (Kelley et al., 2011, 2012). Functional disability leads older adults to require more help and caregiving at home (Ankuda & Levine, 2016; Ankuda et al., 2020), or face unmet caregiving needs which itself drives health care use (Depalma et al., 2013). Declines in function have been demonstrated to be more predictive of hospitalization in seriously ill patients than stable disability itself (Kelley et al., 2012). However, while the Improving Medicare Post-Acute Care Transformation Act mandates screening for disability in postacute care settings, hospital and outpatient settings do not routinely screen for physical function outside of geriatric specialty practices (Hawes et al., 1995; Osakwe et al., 2017).

The most common measure of functional disability and decline are the ADLs. First described by Katz, ADL disabilities were identified through observing the sequential loss of domains of ability of older adults over time. They originally contained the six functions of bathing, dressing, toileting, transferring, continence, and feeding (Katz et al., 1963), with mobility added to later versions.

Life space constriction (LSC) considers the frequency with which individuals leave their proximal environments and therefore provides a broader perspective of disability (Baker et al., 2003). Life space can be conceptualized as concentric rings of the spaces within which individuals move, from the room in which one sleeps, to the neighborhood, to the entire world (Webber et al., 2010). LSC can be measured with a single-item assessment (Laffan, 2005; Stalvey et al., 1999; Xue et al., 2008) and is correlated with risk of death (Boyle et al., 2010; Kennedy et al., 2017; Mackey et al., 2014, 2016; Xue et al., 2008). Those with severe LSC, who rarely or never leave home and are referred to as being homebound (Ornstein et al., 2015), have high levels of mortality and health care use (Musich et al., 2015; Norman et al., 2018; Reckrey et al., 2013; Soones et al., 2017).

It is important to assess the independent and cumulative risks of LSC and ADL impairment because disability is the result of a gap between one’s physical/cognitive abilities and environmental and personal supports (Verbrugge & Jette, 1994). Therefore, we hypothesize that LSC captures equally important yet distinct consequences of disability as compared to ADLs alone. For example, two individuals with the same ADL disability may vary considerably in their ability to socialize, work, and go to religious services because of differences in their finances, caregiver support, or built environments. While both LSC and ADLs have separately been demonstrated to be associated with death and hospitalization, it is unclear how LSC compares or adds to ADL disability screening to predict population health outcomes.

Given the importance of health system screening for functional disability, we aim to examine the prognostic characteristics of population ADL disability versus LSC screening. Specifically, we aim to examine how LSC performs separate from and in combination with ADL disabilities in predicting hospitalization and mortality and to characterize the populations who would be identified by ADL disability versus LSC screens. We hypothesize that distinct populations will be identified by ADL versus LSC screens, and these measures together will be more predictive of mortality and hospitalization than either measure separately. This work will inform future population health efforts to screen for high-need patients with functional disability and decline.

## Method

### Study Sample

We used data from the National Health and Aging Trends Study (NHATS), a nationally representative survey of Americans aged 65 and older that began in 2011. The sample was drawn from the Medicare enrollment file with oversamples at older ages and of Black enrollees. The survey has been administered annually with a response rate of 71% to for the initial round that individuals entered NHATS (in 2011), and from 86.1% to 94.8% for follow-up surveys (Montaquila et al., 2011). NHATS oversamples persons at older ages and Black individuals to allow for subgroup estimates by age and race, and provides survey weights to adjust analyses to be nationally representative (Freedman et al., 2020). NHATS asks about a variety of health, disability, individual, and household factors. In addition, NHATS is linked to the Medicare Master Beneficiary Summary File, allowing identification of deceased individuals. NHATS conducts surveys with proxy reporters if the participant cannot respond, such as if they are too ill, or have a speech or hearing impairment. For additional details on proxy respondents, see Supplementary Table S4. The NHATS is sponsored by the National Institute on Aging (grant number NIA U01AG032947) through a cooperative agreement with the Johns Hopkins Bloomberg School of Public Health.

### Cohort

We included respondents from NHATS Waves 1–6 (2011–2016). We limited the sample to individuals who entered NHATS in 2011, with individual respondents observed multiple times. We excluded the 2,798 observations that had missing data for either ADLs or LSC (8.3% of the sample). Given that seven annual waves have been conducted, this allowed us to look forward to the next survey wave for each observation to assess for death and hospitalization.

### Functional Disability Measures

ADLs were defined as the total number of activities that the participant reported receiving assistance with or not doing in the last month: bathing, eating, dressing, toileting, getting out of bed, and walking inside (Katz et al., 1963). ADL disabilities were categorized as none, one to two, or three to six, given other work demonstrating that these are reasonable clinical categories of ADL disability (Depalma et al., 2013; Freedman et al., 2011) as well as limitations on reportable cell sizes of data through NHATS. We also separately considered ADL disability as a binary measure of no ADL disability or one or more ADL disabilities.

LSC can be measured with a single-item assessment (Laffan, 2005; Stalvey et al., 1999; Xue et al., 2008). This is a simpler approach than other LSC assessments that separately assess the frequency and difficulty with which individuals leave each life space (bedroom, house, porch/garage, etc.; McCrone et al., 2019). The NHATS LSC item asks respondents how often they left their home to go outside in the last month: every day, most days (5–6 days per week), some days (2–4 days per week), or rarely/never. We additionally separately considered LSC as a binary measure of leaving the house every day versus less often than every day. This measure has both been validated in NHATS and used in multiple epidemiologic studies (Ornstein et al., 2015; Soones et al., 2017; Szanton et al., 2016; Xiang & Brooks, 2017).

### Outcome Measures

Mortality in the following year was ascertained by NHATS interviewers during annual surveys. Death was reported by informants during attempts to contact the participant for the annual interview, and assessed via the Medicare Beneficiary Summary File. Given that Medicare claims data are not available linked to NHATS for the third of older adults enrolled in Medicare Advantage plans, hospitalization in the last year was self-reported by participants. However, all NHATS enrollees have a linked Medicare Beneficiary Summary File, meaning that data are available for all NHATS respondents.

### Other Measures

In order to capture the sociodemographics and health of populations identified through functional screening in this sample, we used additional variables including: demographic characteristics (sex, age, race); proxy respondent status; socioeconomic characteristics (lives alone, size of social network, not enough money for health care bills and medications, not enough money for utilities and rent, Medicaid insurance); self-reported illness (heart attack, heart disease, hypertension, arthritis, osteoporosis, diabetes, lung disease, stroke, cancer, depression as measured with the Patient Health Questionnaire-two items [PHQ-2]; Arroll et al., 2010; Xiang & Brooks, 2017); cognitive status as defined as probable or possible dementia, measured through a validated algorithm that includes self-report of receipt of a dementia or Alzheimer’s diagnosis, the eight-item AD8 Dementia Screening Interview, and direct cognitive testing (Kasper et al., 2013); and other illness characteristics (self-reported fair or poor health, falling in the last month, bothersome pain in the last month; Kasper & Freedman, 2020).

### Statistical Analysis

We measured the 1-year hospitalization and mortality rates for the entire cohort and for those meeting each definition of ADL and LSC-based functional disability and decline. Estimates were calculated using survey weights that take into account both the sample design of NHATS and differential response probabilities. We then compared predictive models for 1-year mortality. Given that we were interested in testing if ADL and LSC individually improve prediction and how much ADLs and LSC *together* improve prediction over either alone, we build four separate models: (a) a baseline model including just age and gender given that mortality statistics commonly adjust for these factors (National Vital Statistics Center); (b) a model that includes age, gender, and the ADL variable; (c) a model that includes age, gender, and the LSC variable; and (d) a model that includes, age, gender, the ADL variable, and the LSC variable. For each model, the area under the receiver operating characteristic (ROC) curve was reported. For the ADL variable, disability was categorized as the count of total ADL disabilities (six levels). For the LSC variable, disability was categorized as leaving the house every day, most days, sometimes, or rarely/never (four levels). Models did not include additional covariates, including comorbidities, given the existing data that demonstrate that function is a better proxy and surrogate for serious illness than comorbidities (Kelley et al., 2017; Landi et al., 2010). Each model was compared to that with the highest ROC using STATA’s roccomp command, which performs a Wald test of the null hypothesis that the area under the ROC curves are equal (Cleves, 2002). We identified any models for which the null could be rejected with a *p*-value of >.05.

We then compared the characteristics of populations who would be identified through population-level screens of ADL disability and LSC, defined as any degree of ADL disability and any degree of LSC disability, as defined above. As some individuals had multiple observations, in all analyses, we both accounted for clustering at the level of the individual and adjusted for survey weighting and design factors (Freedman et al., 2020). As a sensitivity test, we repeated the entire analysis including only the first observation of each individual. This analysis was conducted with STATA 16.0 software.

## Results

We identified 30,885 observations in the NHATS Waves 1–6 (2011–2016) from 7,897 individuals. After applying survey weights and adjusting for repeated observations for each individual (see *Method*), this represented a population that was primarily female (56.5%), with a mean age of 77.2 years (Table 1). Just under a third (31.6%) of the population lived alone, 5.5% reported no individuals in their social network, 12.8% had Medicaid insurance, 3.3% reported that they did not have enough money for health care bills and medications, and 2.8% reported that they did not have enough money for utilities and rent in the last year. Rates of self-reported medical comorbidities ranged from 11.9% for prior stroke to 68.7% for hypertension. Over half (54.3%) had bothersome levels of pain in the last month, 23.2% reported fair or poor health, and 11.5% fell in the last month.

**Table 1.** Baseline Characteristics of Study Population (*N* = 30,885)

Characteristic	%
Female, %	56.5
Age (mean)	77.2
Race/ethnicity	
White, non-Hispanic	81.8
Black, non-Hispanic	8.3
Hispanic	6.5
Other	3.4
Proxy reporter	8.7
Lives alone	31.6
Number of people in social network	
None	5.5
1	35.2
2+	59.3
In the last year, not enough money for	
Health care bills and medications	3.3
Utilities and rent	2.8
Medicaid insurance	12.8
Self-reported illness prevalence	
Heart disease	21.9
Hypertension	68.7
Diabetes	26.3
Lung disease	19.0
Stroke	11.9
Cancer	29.9
Depression (PHQ-2)	12.7
Anxiety (GAD-2)	10.7
Probable/possible dementia^a^	19.5
Self-reported fair or poor health	23.2
Fall in the last month	11.5
Bothersome level of pain	54.3

*Notes*: GAD-2 = Generalized Anxiety Disorder–2 items; PHQ-2 = Patient Health Questionnaire–2 items. This table captures all observations: Individual respondents may have multiple observations. All means and proportions are adjusted for survey design and sampling strategy and for clustering at the respondent-level.

*Source*: National Health and Aging Trends Study data, Wave 1–6 (2011–2016).

^a^Determined through a combination of self-report and cognitive testing.

**Figure 1.**
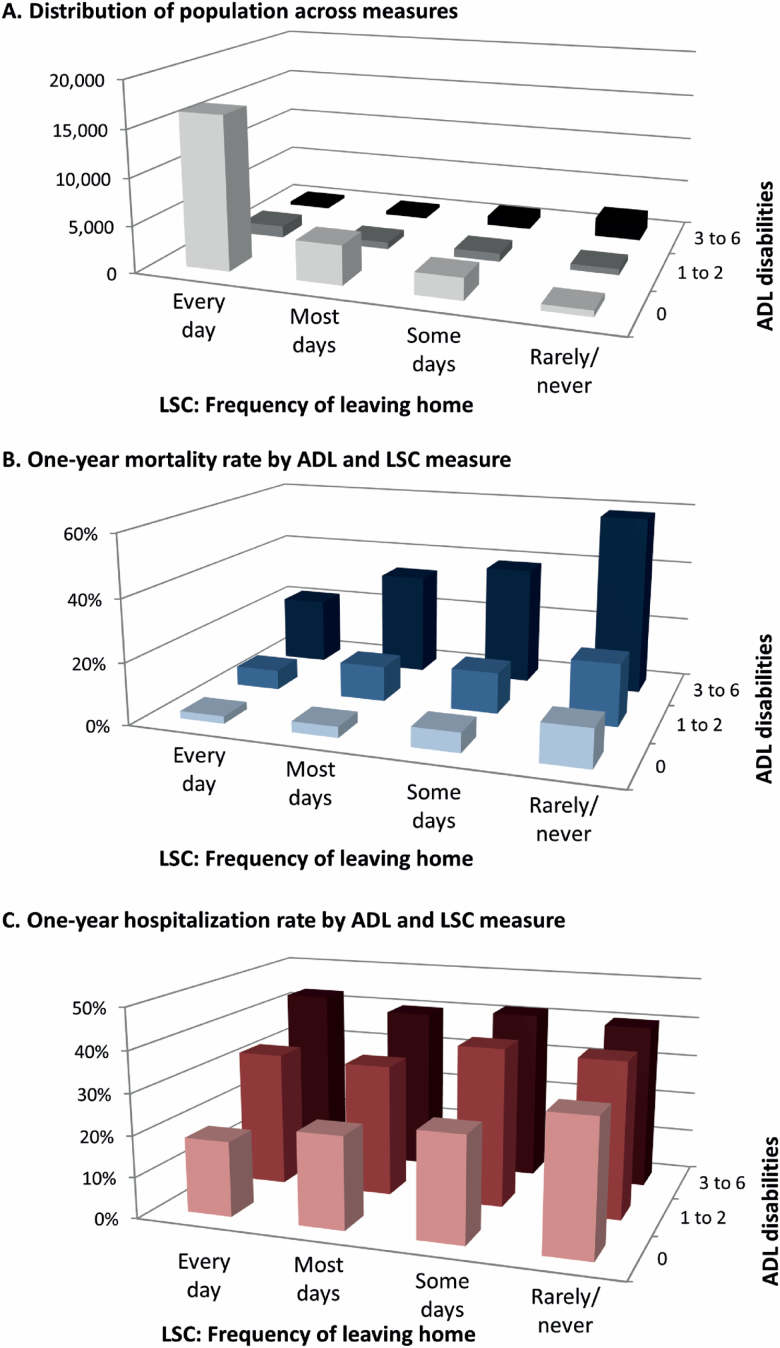
Outcomes by combination of activities of daily living (ADL) disability and life space constriction (LSC). *Note*: 3d representation allows for comparison of population size, mortality rates, and hospitalization rates between each possible combination of LSC and ADL measures. Panel **A** demonstrates the distribution of population across measures. Panel **B** demonstrates the 1-year mortality rate by ADL and LSC measure. Panel **C** demonstrates the 1-year hospitalization rate by ADL and LSC measure.

Figure 1 demonstrates the population distribution along ADL and LSC categories and the comparative rates of hospitalization and mortality for subjects who reported each category of ADL and LSC measures. The proportion of older adults reporting that they rarely/never left the house (10.8%) was roughly similar in size to those with three or more ADL disabilities (12.4%). Individuals reporting disability in ADLs did not always report disability in LSC and vice versa: Figure 1 demonstrates that of the 3,345 subjects who reported they rarely/never left home and the 3,825 subjects who reported three or more ADL disabilities, only 2,049 reported both characteristics. Therefore, about half of persons with disability in three or more ADLs are able to leave their homes several times a week. Of those who rarely/never left home, about 20% had no ADL disability.

Mortality rates increased over categories of ADL disabilities (from 2.9% 1-year mortality for those with no ADL disability to 40.7% for those with three or more ADL disabilities) as well as categories of LSC (from 3.1% 1-year mortality for those who left home every day to 46.4% for those who rarely/never left home). For those with no ADL disability, 1-year mortality ranged from 2.4% to 12.2% across LSC categories, and for those with no LSC disability, 1-year mortality ranged from 2.4% to 21.6% across ADL categories. Those reporting both rarely/never leaving home *and* having three or more ADL disabilities had the highest level of mortality, with 1-year mortality of 58.4%.

Rates of hospitalization similarly increased along ADL categories (from 19.8% 1-year hospitalization for those with no ADL disability to 41.0% for those with three or more ADL disabilities) as well as categories of LSC (from 19.4% 1-year hospitalization for those who left home every day to 37.0% for those who rarely/never left home). The additive effect of ADL disability and LSC measures in predicting hospitalization was less strong than for mortality: the highest hospitalization rate (41.0%) was seen in the population with three or more ADL disabilities who left home. Indeed, among those with three or more ADL disabilities, hospitalization rates were similar across LSC categories (range of 40.0% for those leaving home most days to 42.9% for those leaving home every day). However, among those with no ADL disability, LSC was a strong predictor of hospitalization: within this population, 18.2% of those leaving home every day were hospitalized in the next year, as compared to 32.2% of those who left home rarely/never. For full details of the mortality and hospitalization rates for each combination of ADL and LSC, see Supplementary Table S3.

Table 2 describes the predictive validity as well as number of items for each ADL and LSC category. First, a baseline model including only age and gender predicted 1-year mortality with an area under the ROC curve (aROC) of 0.74 and 1-year hospitalization with an aROC of 0.57. The model with the highest aROC included both ADL disabilities and LSC, with an aROC of 0.86 for mortality and 0.61 for hospitalization. This model performed better than all others, with an aROC that was significantly higher (*p* < .05). All functional disability measures performed better than the baseline model. Our sensitivity test that included only the first observation of each individual did not differ from the primary analysis (see Supplementary Tables S1 and S2).

**Table 2.** Comparison of Predictive Validity for Death and Hospitalization for LSC and ADL-Based Measures

Measure	Area under ROC: 1-year mortality	Area under ROC: 1-year hospitalization
Baseline model	0.74	0.57
Baseline + ADL	0.85	0.60
Baseline + LSC	0.83	0.60
Baseline + ADL + LSC	0.86^a^	0.61^a^

*Notes*: ADL = activities of daily living; LSC = life space constriction; ROC = receiver operator curve. Baseline model includes age and gender. ADLs were categorized as the count of ADLs the individual received help for (bathing, toileting, eating, dressing, transferring in/out of bed, walking inside) with a range of 1–6. LSC was categorized as leaving home every day, most days, sometimes, or rarely/never.

^a^Area under the ROC is significantly higher than that for all other models (*p* < .05).

Table 3 demonstrates the characteristics of those with any degree of ADL disability (defined one or more ADL disabilities) or LSC disability (defined as leaving home less often than every day). Given these are not mutually exclusive classifications, a direct comparison of descriptive characteristics is not possible. Thus, we calculated 95% confidence intervals given the distribution and survey sampling design. We identified characteristics with no overlap in the 95% confidence intervals for those with ADL disability and LSC. Those with LSC were more likely to be female (72.6% vs 63.8% for those with ADL disability), to be Black, non-Hispanic (12.7% vs 8.3%) or Hispanic (11.4% vs 6.5%), and to live alone (40.7% vs 30.7%). Those with LSC were less likely to have a proxy reporter (30.4% vs 35.9% for those with ADL disability), to have dementia (44.7% vs 50.6%) or cancer (27.6% vs 32.4%), to have fallen in the last month (18.2% vs 24.3%) or have had bothersome levels of pain (67.1% vs 70.0%).

**Table 3.** Characteristics of Populations With ADL vs LSC Disability^a^

Characteristic	Any ADL disability, % (95% CI)	Any LSC disability, % (95% CI)
Proportion of cohort	19.2%	19.6%
Any ADL disability	100%	57.0 (55.0–59.0)
Any LCS disability	58.1 (56.2–60.1)	100%
Gender		
Female	63.8 (61.4–66.1)	72.6 (70.5–74.6)^c^
Age		
65–74	23.9 (21.7–26.3)	24.6 (22.5–26.9)
75–84	39.0 (36.9–41.3)	37.9 (35.8–40.0)
>85	37.1 (35.0–39.2)	37.5 (35.4–39.6)
Race		
White, non-Hispanic	76.2 (74.2–78.2)	71.7 (69.5–73.7)^c^
Black, non-Hispanic	8.3 (7.8–8.8)	12.7 (11.6–13.9)^c^
Hispanic	6.5 (5.8–7.3)	11.4 (9.8–13.2)^c^
Other	3.4 (2.9–4.1)	4.3 (3.3–5.6)
Proxy reporter	35.9 (34.0–37.8)	30.4 (28.6–32.1)^c^
Lives alone	30.7 (28.5–33.0)	40.7 (38.3–43.1)^c^
Number of people in social network		
None	8.6 (7.6–9.7)	10.0 (8.8–11.4)
1	33.1 (31.2–35.1)	34.3 (32.4–36.3)
2+	58.3 (56.1–60.4)	55.7 (53.6–57.9)
In the last year, not enough money for		
Health care bills and medications	5.6 (4.6–6.9)	5.5 (4.4–6.7)
Utilities and rent	4.3 (3.5–5.3)	4.2 (3.4–5.3)
Medicaid insurance	27.0 (24.8–29.4)	28.3 (26.2–30.6)
Self-reported illness prevalence		
Heart disease	33.0 (30.6–35.5)	31.2 (29.0–33.6)
Diabetes	36.1 (33.5–38.8)	36.7 (34.2–39.3)
Lung disease	27.0 (24.7–29.4)	27.3 (25.0–29.7)
Stroke	25.7 (23.5–28.1)	22.3 (20.4–24.5)
Cancer	32.4 (30.0–34.9)	27.6 (25.4–29.8)^c^
Depression (PHQ-2)	30.0 (28.2–31.8)	28.9 (27.2–30.7)
Anxiety (GAD-2)	24.3 (22.6–26.1)	23.4 (21.8–25.1)
Probable/possible dementia^b^	50.6 (48.2–53.0)	44.7 (42.5–47.0)^c^
Self-reported fair or poor health	49.5 (47.4–51.6)	49.0 (46.9–51.1)
Fall in the last month	24.3 (22.8–25.8)	18.2 (16.9–19.6)^c^
Bothersome level of pain	70.0 (68.1–71.9)	67.1 (65.2–69.0)

*Notes*: ADL = activities of daily living; CI = confidence interval; GAD-2 = Generalized Anxiety Disorder–2 items; LSC = life space constriction; PHQ-2 = Patient Health Questionnaire–2 items.

^a^ADL disability is defined as receiving help in any ADL, and LSC disability is defined as leaving the house less often than every day. ^b^Determined through a combination of self-report and cognitive testing. ^c^Nonoverlapping 95% CIs in the rate of each characteristic for those with ADL vs LSC disability. Sample proportions are adjusted by survey weights to account for survey sampling and design structure.

## Discussion

Both ADL and LSC screening measures detect a population with high risk of hospitalization and mortality and using both screens together is most predictive of mortality, with 58.4% of those with both disability in three or more ADLs and rarely/never leaving home dying in the next year. Thus, health systems interested in implementing functional disability screening in order to proactively identify those at highest risk of mortality to identify unmet care needs must balance the benefit of screenings versus the time and burden of conducting screens. Health systems with minimal capability to conduct extensive screens could use only the brief LSC, and those who are able to conduct lengthier screens would benefit from implementing both LSC and ADL screens.

This study also demonstrates that ADL and LSC screens capture somewhat distinct clinical populations. While an ADL screen identified more older adults who had a proxy reporter (likely indicating higher levels of cognitive impairment or severe illness), cancer, pain, and falls; those identified with LSC were socially distinct—they are more likely to be female and to live alone. Given the specific risks of loneliness (Perissinotto et al., 2012) and homebound or semihomebound status (Ornstein et al., 2015; Wajnberg et al., 2013), this raises the question of whether the populations identified through LSC versus ADL disability have different service needs. While both methods of screening predict mortality and hospitalization risk equally well, LSC may better identify socially high-risk patients while ADL disability may identify medically high-risk patients, and both together capture the highest-need patients. Further work is needed to understand the distinct experiences and service needs of populations identified through ADL disability versus LSC screens. The population without ADL disability but who rarely/never leave home is notable: it is possible that they face environmental barriers such as unsafe sidewalks that in combination with more mild physical impairments such as inability to walk many blocks or a fear of falling with walking long distances leave them homebound. Alternatively, it is possible that psychiatric or psychological morbidity contributes to their LSC.

We examined two critical outcomes: death and hospitalization. While our results are statistically significant, it is challenging to interpret the clinical significance of comparisons of ROCs. For this reason, we have highlighted the rates of death and hospitalization for each combination of ADL and LSC measure (Figure 1). However, we do not assess patient experience, morbidity, and overall health care utilization following different definitions of functional disability. In addition, our measures of depression and pain may be biased due to high rates of proxy reporters for individuals with severe disability. We also relied on self-reported hospitalization for respondents in Medicare Advantage who did not have linked claims data, which may be prone to measurement error more than a claims-based outcome measure, particularly for those with severe disability more likely to rely on proxy reporters for NHATS. This may explain why these functional measures were less predictive of hospitalization than mortality, which is less subject to measurement bias. In addition, as it is retrospectively reported, we cannot capture hospitalizations for those who died in the year following the interview assessing disability. This may explain why LSC and ADL disability were less predictive of hospitalization. Further longitudinal analyses and qualitative work with finer-grained data on the temporal relationship between different disability assessments and outcomes are needed to understand how disability predicts health care use. In addition, further work needs to understand the role of cognitive impairment and dementia in shaping both risks for LSC and ADL disability.

The ability to leave home and function in social roles, as LSC captures, is extraordinarily important to people (Carruth, 2011; Fried et al., 2011; Kenyon, 1996). This study demonstrates that LSC independently predicts mortality and hospitalization and improves on the ability of ADLs alone to predict both outcomes. While both of these screening tools identify high-risk, functionally impaired populations that largely overlap, their distinct demographic and social characteristics suggest these groups may have different care needs. This work provides a platform for future research comparing the experience and quality of life of older adults with ADL versus LSC disability as well as best approaches to incorporating both markers, including testing more complex models, interactions between disability types, and moderators such as cognitive status. Further work should also examine the full spectrum of LSC: from mobility inside the home, to that within the proximal neighborhood and broader town or city. As health care systems attempt to target high-risk patient populations, including those with functional decline, it will be critical to understand the longitudinal trajectories and care needs of these populations identified through distinct functional disability screens (American Academy of Hospice and Palliative Medicine, n.d.; Coalition to Transform Advanced Care, 2017). Future work refining the use of screening tools to identify populations with specific care needs will help to efficiently target palliative care and geriatric interventions and other high-resource programs to the people most likely to benefit. Evidence from health system screenings will be important to also more accurately assess the risk–benefit of functional disability screening that also considers the time and burden of implementing screens for disability and the potential services that a given system can deliver as a result of a positive screen. Improving our understanding of the performance of functional decline screens is critical for health systems to comprehensively improve the quality of care for high-risk older adults.

## Supplementary Material

igaa065_suppl_Supplementary_Materials
