# Supplementary material for: Population-Based Screening for Functional Disability in Older Adults
Source: Innov Aging. 2020 Dec 22;5(1):igaa065. doi: 10.1093/geroni/igaa065 (PMC7817111; doi:10.1093/geroni/igaa065)
Supplement: igaa065_suppl_Supplementary_Materials [file igaa065_suppl_supplementary_materials.docx]

Online Supplementary Material for Publication in *Innovation in Aging*:

Population-based screening for functional disability in older adults

Claire K. Ankuda, MD, MPH^1^ , Vicki A. Freedman, PhD^2^, Kenneth E. Covinsky, MD, MPH^3, 4^, Amy S. Kelley, MD, MSHS^1,5^

^1^Department of Geriatrics and Palliative Medicine, Icahn School of Medicine at Mount Sinai, New York, New York, USA

^2^Institute for Social Research, University of Michigan, Ann Arbor, Michigan, USA

^3^Division of Geriatrics, University of California, San Francisco, San Francisco, California, USA

^4^Division of Geriatrics, Veterans Affairs Medical Center, San Francisco, California, USA

^5^Geriatric Research Education and Clinical Center, James J. Peters Veterans Affairs Medical Center, Bronx, New York, USA

*Address correspondence to: Claire K. Ankuda, MD, MPH, One Gustave L. Levy Place, Box 1070, New York, NY 10029 USA. E-mail: [claire.ankuda@mssm.edu](mailto:claire.ankuda@mssm.edu)

| **Supplementary Materials Table S1. Baseline Characteristics of First Observation of Study Population, Limited to First Observation of Each Individual (N=11,882).** | |
| --- | --- |
| *Characteristic* | *%* |
| Female, % | 55.7 |
| Age (mean) | 74.2 |
| Race/ethnicity |  |
| White, non-Hispanic | 80.7 |
| Black, non-Hispanic | 8.2 |
| Hispanic | 7.2 |
| Other | 3.9 |
| Proxy reporter | 6.0 |
| Lives alone | 29.0 |
| Number of people in social network |  |
| None | 5.8 |
| 1 | 38.1 |
| 2+ | 56.1 |
| In the last year, not enough money for |  |
| Healthcare bills and medications | 4.4 |
| Utilities and rent | 4.4 |
| Medicaid insurance | 12.0 |
| Self-reported illness prevalence |  |
| Heart disease | 16.0 |
| Hypertension | 63.1 |
| Diabetes | 24.5 |
| Lung disease | 15.3 |
| Stroke | 8.9 |
| Dementia | 17.9 |
| Cancer | 24.6 |
| Depression (PHQ-2) | 13.6 |
| Anxiety (GAD-2) | 11.8 |
| Self-reported fair or poor health | 23.6 |
| Fall in the last month | 10.1 |
| Bothersome level of pain | 53.0 |
| Source: NHATs data, wave 1-6 (2011-2016).  *Notes.* All means and proportions are adjusted for survey design and sampling strategy. PHQ-2= Patient Health Questionnaire-2 item, GAD-2= Generalized Anxiety Disorder-2 item. | |

| **Supplementary Materials Table S2. Comparison of Predictive Validity for Death and Hospitalization for LSC and ADL-Based Measures, Limited to First Observation of Each Individual.** | | |
| --- | --- | --- |
| Measure | Area under ROC: 1-year mortality | Area under ROC: 1-year hospitalization |
| *Baseline model* | *0.79* | *0.57* |
| Baseline + ADL | 0.87 | 0.60 |
| Baseline + LSC | 0.85 | 0.60 |
| Baseline + ADL + LSC | 0.88* | 0.61* |
| *Notes*. baseline model includes age and gender. ADL= activities of daily living, LSC= life space construction, ROC= receiver operator curve.  * = Area under the ROC is significantly higher than that for all other models (p<0.05). | | |

| **Supplementary Materials Table S3. Outcomes by Combination of Activity Daily Living (ADL) Disability and Life Space Constriction (LSC).** | | | | | |
| --- | --- | --- | --- | --- | --- |
| A. Population size and distribution | | | | | |
|  |  | ADL impairments | | |  |
|  |  | 0 | 1 to 2 | 3 to 6 |  |
| How often leaves home | Every day | 16,365 | 1,263 | 372 | 18,000 (58.3%) |
|  | Most days | 4,185 | 732 | 390 | 5,307 (17.2%) |
|  | Some days | 2,331 | 888 | 1,014 | 4,233 (13.7%) |
|  | Rarely/never | 654 | 642 | 2,049 | 3,345 (10.8%) |
|  |  | 23,535 (76.2%) | 3,525 (11.4%) | 3,825 (12.4%) |  |
|  | | | | | |
| B. One-year mortality rate by ADL and LSC measure | | | | | |
|  |  | ADL impairments | | |  |
|  |  | 0 | 1 to 2 | 3 to 6 |  |
| How often leaves home | Every day | 2.4% | 6.4% | 21.6% | 2.9% |
|  | Most days | 3.5% | 11.5% | 33.2% | 6.3% |
|  | Some days | 6.3% | 13.3% | 38.5% | 14.6% |
|  | Rarely/never | 12.2% | 20.1% | 58.4% | 40.7% |
|  |  | 3.1% | 11.3% | 46.4% |  |
|  | | | | | |
| C. One-year hospitalization rate by ADL and LSC measure | | | | | |
|  |  | ADL impairments | | |  |
|  |  | 0 | 1 to 2 | 3 to 6 |  |
| How often leaves home | Every day | 18.2% | 32.8% | 42.9% | 19.4% |
|  | Most days | 22.4% | 32.1% | 40.0% | 24.3% |
|  | Some days | 25.5% | 38.6% | 41.5% | 30.7% |
|  | Rarely/never | 32.2% | 37.8% | 40.2% | 37.0% |
|  |  | 19.8% | 34.7% | 41.0% |  |
|  | | | | | |
| Source: National Health and Aging Trends Study (NHATs), 2011-2016.  *Notes.* ADL: difficulty or relying on assistance in activities of daily living (walking inside, walking outside, transferring in/out of bed, bathing, toileting, eating, dressing). LSC: life space construction. ^1^Death and hospitalization are not mutually exclusive outcomes. | | | | | |

| **Supplementary Table S4: % With Proxy Respondents by Functional Measure** | |
| --- | --- |
| Functional measures | % With proxy respondents |
| Number of ADLs: |  |
| 0 | 2.1% |
| 1 | 9.6% |
| 2 | 17.7% |
| 3 | 29.3% |
| 4 | 48.2% |
| 5 | 62.7% |
| 6 | 82.5% |
| How often leaves home: |  |
| Every day | 2.5% |
| Most days | 5.8% |
| Some days | 15.5% |
| Rarely/never | 46.1% |
| *Notes.* ADLs= Activities of daily living that a respondent receives help with: bathing, transferring in/out of bed, toileting, eating, dressing, walking inside | |
